# Earthquake preparedness and earthquake-related anxiety among emergency department patients: a cross-sectional study

**DOI:** 10.1515/med-2026-1537

**Published:** 2026-09-21

**Authors:** Burak Demirci, Burak Akın, Enes Ferhatlar, Selman Gündoğan, Ömer Kilim, Abuzer Coskun

**Affiliations:** Department of Emergency Medicine, Istanbul Bağcılar Training and Research Hospital, Health Sciences University, Istanbul, Türkiye

**Keywords:** earthquake preparedness, disaster preparedness, earthquake-related anxiety, emergency department, preparedness behavior, disaster risk perception

## Abstract

**Objectives:**

To describe earthquake-preparedness behaviors and earthquake-related anxiety responses in emergency department patients and examine associated factors and the relationship between the two composites.

**Methods:**

This cross-sectional study included 3,964 adults presenting to an emergency department in an earthquake-prone region between July and December 2025. A structured questionnaire assessed sociodemographic and housing characteristics, 15 binary preparedness behaviors, and five earthquake-related anxiety items. Preparedness was summarized as a behavioral composite (0–15) and anxiety as an exploratory composite (5–25). Nonparametric tests, effect sizes, false discovery rate adjustment, exploratory internal-structure analyses, and multivariable regression were used.

**Results:**

Median preparedness was 9 (IQR, 7–12) and median anxiety was 13 (IQR, 7–18). Knowledge-based behaviors were common, whereas practical measures were less frequent. Most group differences had small effect sizes. Preparedness showed a multidimensional structure, whereas anxiety showed a single-dimension exploratory structure. The inverse correlation was very weak (Spearman’s ρ=−0.107; 95 % CI, −0.140 to −0.070). After adjustment, higher anxiety was associated with lower preparedness (B=−0.074; 95 % CI, −0.090 to −0.059), and higher preparedness with lower anxiety (B=−0.241; 95 % CI, −0.289 to −0.193).

**Conclusions:**

Practical preparedness remained incomplete. Preparedness and anxiety were weakly associated; findings are exploratory and should not be interpreted causally or diagnostically.

## Introduction

Earthquakes remain among the most destructive natural hazards worldwide, causing extensive mortality, injury, displacement, infrastructure damage, and long-term societal disruption. In densely populated metropolitan regions located near active fault systems, the consequences of a major earthquake may be amplified by structural vulnerability, population density, and the increased burden placed on healthcare systems. Although earthquakes cannot be prevented, their human and societal impact can be substantially reduced through effective disaster risk reduction and preparedness strategies [1].

Disaster preparedness refers to the actions undertaken by individuals and households to anticipate, respond to, and cope with hazardous events. These actions may include awareness of structural safety, securing household furniture, preparing emergency supply kits, identifying safe assembly areas, and establishing family emergency plans. Previous research has demonstrated that preparedness behaviors are influenced by multiple social, cognitive, and environmental determinants, including perceived risk, access to information, education level, and prior exposure to disaster-related knowledge [2], [3], [4]. In this context, preparedness is better understood as a set of distinct knowledge-based and practical behaviors rather than as a single uniform construct.

Despite increasing public awareness of disaster risk in many earthquake-prone regions, a persistent challenge remains the gap between risk awareness and actual preparedness behavior. Individuals may acknowledge the possibility of a disaster but still fail to implement practical preparedness measures in their daily lives. Studies examining household disaster readiness have repeatedly shown that awareness alone is insufficient to ensure concrete preparedness actions [5], 6]. This distinction is particularly relevant when preparedness is evaluated through observable household practices rather than through a single standardized scale.

Behavioral models have further suggested that preparedness behaviors are shaped not only by information exposure but also by psychological constructs such as perceived vulnerability, self-efficacy, perceived control, and beliefs regarding the effectiveness of protective actions. Evidence from behavioral research indicates that preparedness interventions are most effective when they address both cognitive and psychological factors associated with protective behavior rather than relying solely on risk communication [7].

In addition to practical preparedness, disaster risk also has an important psychological dimension. Living in an earthquake-prone environment may generate anticipatory fear, sleep disturbances, hypervigilance, and persistent disaster-related anxiety even in the absence of a recent catastrophic event. Large-scale disaster mental health research has demonstrated that threat perception and psychological distress play a central role in shaping how individuals interpret and respond to disaster risk [8].

The relationship between preparedness and anxiety, however, is complex. Moderate levels of concern may be associated with greater engagement in protective behaviors, whereas higher levels of fear may coexist with avoidance, helplessness, or fatalistic attitudes toward disaster risk. Examining how earthquake-related anxiety is associated with preparedness behaviors may therefore contribute to a more balanced understanding of disaster-related responses [3], 7], 8].

Healthcare systems, and particularly emergency departments, occupy a critical position within disaster response structures. Emergency departments serve heterogeneous populations representing diverse socioeconomic and educational backgrounds and often function as the first point of healthcare access within urban communities. Consequently, evaluating preparedness behaviors and earthquake-related psychological responses among individuals presenting to emergency departments may provide insight into patterns observed within this accessible and heterogeneous healthcare population [9], 10].

Although disaster preparedness, risk perception, and disaster-related psychological responses have been examined in different populations, fewer studies have evaluated practical preparedness behaviors and earthquake-related anxiety responses together in a large emergency department sample. Emergency department attendees represent a clinically stable but non-random healthcare population with diverse demographic and socioeconomic characteristics. Evaluating these domains together may help characterize the awareness–action gap and the factors associated with preparedness and anxiety within this specific setting.

Therefore, the present study aimed to describe self-reported earthquake-preparedness behaviors and earthquake-related anxiety responses among adults presenting to a tertiary emergency department, to examine demographic, socioeconomic, occupational, and housing-related factors associated with these measures, and to evaluate the relationship between the preparedness and anxiety composites. Given the cross-sectional design and the investigator-developed nature of the composite measures, all findings were interpreted as exploratory associations rather than causal or diagnostic conclusions.

## Materials and methods

### Study design and setting

This cross-sectional observational study was conducted in the emergency department of Kanuni Training and Research Hospital, a tertiary-care institution serving a large and socioeconomically heterogeneous population in a metropolitan region with substantial earthquake risk. Data were collected between 1 July 2025 and 31 December 2025. The primary objective was to describe self-reported earthquake-preparedness behaviors and earthquake-related anxiety responses among adults presenting to the emergency department. The secondary objectives were to identify demographic, socioeconomic, occupational, and housing-related factors associated with these measures and to examine the relationship between preparedness and earthquake-related anxiety.

### Participants and recruitment

Adult patients presenting to the emergency department during the study period were considered eligible if they were aged 18 years or older, clinically stable at the time of recruitment, able to understand and complete the questionnaire, and willing to provide informed consent. Patients with impaired consciousness, severe clinical instability, acute cognitive disturbance, or another condition preventing reliable questionnaire completion were excluded.

Participants were recruited using a non-probability convenience-sampling approach during the predefined data-collection period. The questionnaire was administered after the initial clinical assessment and stabilization of the patient so that participation would not interfere with emergency medical care. Participants completed the questionnaire themselves, and research personnel provided clarification when necessary without directing their responses.

Some eligible individuals declined participation or discontinued the questionnaire because they did not wish to remain in the emergency department longer, had time constraints or urgent personal obligations, were unwilling to complete a questionnaire, or lost interest before completion. The exact number and sociodemographic characteristics of individuals who declined participation or submitted incomplete forms were not systematically recorded. Therefore, participants and nonparticipants could not be compared, and the possibility of nonresponse and selection bias could not be quantified. Questionnaires with incomplete responses to variables required for the principal analyses were not included. The final analytical sample consisted of 3,964 participants with complete data for the variables reported in the present study. No missing-value imputation was performed.

### Sample size considerations

The study was designed as a time-limited survey of accessible and eligible emergency department attendees during the six-month data-collection period. Therefore, the final sample size was determined by the number of eligible individuals who consented to participate and completed the questionnaire during this period rather than by a formal *a priori* power calculation. A retrospective power calculation was not used because such calculations provide limited information once the observed effect estimates and their precision are available. Accordingly, the revised analyses emphasize effect sizes, 95 % confidence intervals, false discovery rate-adjusted p values, and the magnitude of associations rather than statistical significance alone. The absence of a formal *a priori* sample size calculation was considered when interpreting the findings.

### Data-collection instrument

Data were collected using a structured questionnaire developed by the investigators with reference to the practical components of household earthquake preparedness described in the disaster-preparedness literature and with consideration of expert clinical and disaster-management opinion. The questionnaire collected sociodemographic and housing information, self-reported earthquake-preparedness behaviors, and earthquake-related psychological responses.

The sociodemographic and housing section included sex, age group, marital status, household size, occupation, educational attainment, monthly household income, housing tenure, number of floors in the residential building, and building age. Age, education, income, occupation, and housing characteristics were recorded using the categories specified in the questionnaire before analysis.

### Preparedness behavioral composite

Earthquake preparedness was assessed using 15 binary items addressing distinct knowledge-based and behavioral components of preparedness. These items evaluated knowledge of the earthquake resistance of the participant’s residence, fixation of cabinets and shelves, preparation of a family emergency plan, knowledge of the local emergency assembly point, knowledge of appropriate actions during an earthquake, first-aid knowledge, availability of an emergency disaster kit, regular renewal of the disaster kit, pre-identification of emergency contacts, awareness of regional earthquake risk, availability of emergency equipment at home, preparation of a family communication plan, identification of a post-disaster meeting point, previous participation in disaster-preparedness training, and earthquake insurance coverage.

Each affirmative response was assigned a value of 1 and each negative response a value of 0. The item values were summed to obtain a composite count ranging from 0 to 15, with higher values indicating a greater number of reported preparedness behaviors. This measure was not developed or used as a diagnostic instrument, validated psychometric scale, predictive score, or newly proposed scoring system. Because the items address heterogeneous and non-interchangeable preparedness behaviors, the total was interpreted as a pragmatic behavioral checklist composite used to summarize reported preparedness practices within this study.

The questionnaire initially included descriptive categories corresponding to low, moderate, and high preparedness. These categories were created by dividing the possible range into three intervals and were not based on externally validated thresholds. To avoid treating these cut points as established classifications, they were not used in the principal analyses. The continuous composite count was used for correlation and multivariable modeling.

### Earthquake-related anxiety composite

Earthquake-related anxiety responses were assessed using five investigator-developed Likert-type items. The items addressed anxiety when thinking about earthquakes, fear that an earthquake might occur during sleep, perceived loss of control during an earthquake, avoidance of earthquake-related news or content, and consideration of moving because of earthquake risk. Each item was rated from 1, indicating strong disagreement, to 5, indicating strong agreement.

The five responses were summed to obtain an exploratory composite ranging from 5 to 25, with higher values indicating stronger earthquake-related anxiety responses. This composite was used to summarize the responses collected in this study and was not intended to diagnose an anxiety disorder or replace a validated psychiatric instrument. No externally validated clinical thresholds were applied, and the continuous composite was used in the principal analyses.

### Internal consistency and exploratory internal-structure analyses

Internal consistency and exploratory internal structure were examined to characterize the behavior of the investigator-developed composites within the present sample. These analyses were undertaken because the measures had not undergone prior external validation and were not interpreted as formal validation of a new scale. Cronbach’s alpha, corrected item–total correlations, and Cronbach’s alpha after deletion of each item were calculated for both composites. Sampling adequacy was evaluated using the Kaiser–Meyer–Olkin statistic, and Bartlett’s test of sphericity was used to assess the suitability of the observed item-correlation matrices for exploratory dimensionality analysis.

Exploratory internal-structure analyses were performed separately for the 15 binary preparedness items and the five Likert-type earthquake-related anxiety items using the observed item-correlation matrices. The number of dimensions to retain was determined by parallel analysis, in which observed eigenvalues were compared with the 95th-percentile eigenvalues obtained from randomly generated datasets with the same sample size and number of items. Components exceeding the corresponding simulated eigenvalues were retained. For the preparedness items, the retained multidimensional solution was subjected to orthogonal rotation to facilitate interpretation of the loading pattern. Rotation was not required for the anxiety composite because parallel analysis supported a single dimension. The number of retained dimensions, cumulative proportion of variance explained, and range of the largest absolute item loadings across the retained dimensions were reported.

Because the preparedness items represented conceptually distinct forms of knowledge, planning, equipment ownership, training, communication, and financial protection, a multidimensional solution was considered substantively plausible. The analyses were used only to characterize internal structure within the present dataset and were not considered evidence of diagnostic validity, criterion validity, test–retest reliability, external validity, or generalizability.

### Statistical analysis

Categorical variables were summarized as number and percentage. Continuous variables and composite measures were summarized using mean and standard deviation together with median and interquartile range. Distributional characteristics of the preparedness and earthquake-related anxiety composites were examined before inferential analyses. Because these measures were bounded and did not demonstrate distributions appropriate for conventional parametric group comparisons, nonparametric methods were used for the unadjusted analyses.

Comparisons between two independent groups were performed using the Mann–Whitney U test, whereas comparisons involving more than two groups were performed using the Kruskal–Wallis test. Rank-biserial correlation was reported as the effect-size measure for two-group comparisons, and epsilon-squared was reported for comparisons involving more than two groups. The magnitude of the effect sizes was considered together with statistical significance because the large sample size could produce small p values for associations of limited magnitude.

Associations between each of the 15 reported preparedness behaviors and the earthquake-related anxiety composite were examined using the Mann–Whitney U test, with rank-biserial correlation reported as the corresponding effect size. To control the expected proportion of false-positive findings arising from multiple exploratory comparisons, the Benjamini–Hochberg false discovery rate procedure was applied separately to the relevant families of hypothesis tests. Both unadjusted and FDR-adjusted p values were reported so that the original comparisons and the additional multiplicity control remained transparent.

The relationship between the continuous preparedness and earthquake-related anxiety composites was evaluated using Spearman’s rank correlation coefficient. A 95 % confidence interval for Spearman’s rho was estimated using bootstrap resampling. The strength of the correlation was interpreted according to its magnitude rather than its p value alone.

Two multivariable regression models were constructed to evaluate adjusted associations. In Model A, the preparedness composite was entered as the dependent variable, and the earthquake-related anxiety composite was included as the primary explanatory variable. In Model B, the earthquake-related anxiety composite was entered as the dependent variable, and the preparedness composite was included as the primary explanatory variable. Both models simultaneously included sex, age group, marital status, household size, housing tenure, occupation, educational attainment, monthly income, number of building floors, and building age. These covariates were selected before interpretation of the multivariable results because of their plausible relationships with socioeconomic resources, residential vulnerability, preparedness opportunities, and psychological responses to earthquake risk.

Categorical predictors were entered using indicator variables, with reference categories stated in Table 6. Household size, number of building floors, building age, and the two composite measures were modeled continuously. Unstandardized regression coefficients were reported with heteroscedasticity-robust HC3 standard errors, 95 % confidence intervals, and two-sided p values. Robust standard errors were used to reduce sensitivity of inference to nonconstant residual variance. Model performance was summarized using R^2^ and adjusted R^2^. Regression findings were interpreted as adjusted associations only; the cross-sectional design does not establish temporal order or causality in either direction.

Complete-case analysis was used because the final analytical dataset contained complete responses for the variables included in the reported models. No statistical imputation was undertaken. Statistical significance was defined as a two-sided p-value below 0.05, while effect sizes, confidence intervals, and FDR-adjusted results were given priority in interpretation.

The primary data management and conventional statistical analyses were performed using IBM SPSS Statistics, version 26.0. Additional analyses required for the revision, including Benjamini–Hochberg false discovery rate adjustment, bootstrap confidence intervals, parallel analysis, exploratory internal-structure analyses, and heteroscedasticity-robust HC3 regression inference, were performed using Python-based statistical computing procedures. The analyses used the same final complete-case dataset of 3,964 participants as the primary analyses.

#### Ethical considerations

The study protocol was approved by the Clinical Research Ethics Committee of Kanuni Training and Research Hospital on 11 June 2025, with decision number 142. The study was conducted in accordance with the principles of the Declaration of Helsinki. Participation was voluntary, responses were collected anonymously, and no personally identifiable information was recorded.

## Results

### Study population and participant characteristics

A total of 3,964 participants with complete data for the variables included in the analyses were evaluated. The study population was predominantly male (66.7 %), and 50.6 % of participants were aged 18–30 years. High school and university graduates constituted 30.3 and 35.5 % of the sample, respectively. Most participants reported a monthly income of ≤75,000 TRY, 52.0 % lived in rented housing, and 49.4 % resided in buildings older than 20 years (Table 1).

**Table 1:** Selected sociodemographic and housing characteristics and their associations with the preparedness composite and earthquake-related anxiety composite.

Participant characteristics			Preparedness composite				Earthquake-related anxiety composite			
Variable	Category	n (%)	Median (IQR)	p-Value	Effect size	FDR-adjusted p-Value	Median (IQR)	p-Value	Effect size	FDR-adjusted p-Value
Gender	Male	2,645 (66.7)	10 (7–13)	<0.001	0.187	<0.001	13 (7–17)	<0.001	−0.137	<0.001
	Female	1,319 (33.3)	9 (7–10)				15 (7–19)			
Age group	18–30 years	2,007 (50.6)	9 (7–11)	<0.001	0.023	<0.001	14 (11–18)	<0.001	0.115	<0.001
	30–40 years	1,025 (25.9)	9 (7–12)				8 (5–18)			
	40–50 years	468 (11.8)	11 (5–13)				19 (12–23)			
	50–60 years	242 (6.1)	10 (9–11)				11 (7–12)			
	60–70 years	136 (3.4)	11 (10–12)				6 (5–7)			
	≥70 years	86 (2.2)	7 (7–8)				19 (10–20)			
Education	No education	58 (1.5)	3 (3–7)	<0.001	0.039	<0.001	22 (19–23)	<0.001	0.086	<0.001
	Primary	544 (13.7)	8 (7–11)				14 (5–25)			
	Secondary	505 (12.7)	10 (9–11)				7 (5–15)			
	High school	1,203 (30.3)	9 (6–11)				13 (9–20)			
	University	1,408 (35.5)	10 (7–12)				14 (11–18)			
	Postgraduate	246 (6.2)	13 (7–13)				10 (10–19)			
Income, TRY	0–25,000	1,660 (41.9)	9 (7–11)	<0.001	0.041	<0.001	12 (5–18)	<0.001	0.025	<0.001
	25–50,000	1,027 (25.9)	9 (8–11)				15 (10–18)			
	50–75,000	851 (21.5)	10 (6–13)				15 (9–19)			
	75–100,000	155 (3.9)	14 (11–14)				14 (11–14)			
	100–150,000	240 (6.1)	10 (7–13)				13 (10–21)			
	>150,000	31 (0.8)	12 (12–12)				12 (12–12)			
Housing status	Rent	2,062 (52.0)	9 (7–11)	<0.001	−0.124	<0.001	14 (5–18)	0.091	−0.031	0.097
	Owner	1,902 (48.0)	10 (7–13)				13 (10–17)			
Number of floors	0–5 floors	2,647 (66.8)	9 (7–11)	<0.001	0.043	<0.001	14 (7–18)	<0.001	0.017	<0.001
	6–10 floors	765 (19.3)	8 (6–11)				13 (5–17)			
	>10 floors	552 (13.9)	12 (9–14)				14 (12–16)			
Building age	0–10 years	1,117 (28.2)	10 (9–13)	<0.001	0.049	<0.001	13 (10–17)	0.004	0.002	0.005
	10–20 years	890 (22.5)	9 (6–11)				11 (7–20)			
	>20 years	1,957 (49.4)	9 (7–11)				14 (7–18)			

Data are presented as n (%) for categorical variables and median (interquartile range, IQR) for the preparedness composite and earthquake-related anxiety composite. Comparisons between two groups were performed using the Mann–Whitney U test, and comparisons involving more than two groups were performed using the Kruskal–Wallis test. Effect sizes are reported as rank-biserial correlation for two-group comparisons and epsilon-squared for comparisons involving more than two groups. To account for multiple hypothesis testing, p values were additionally adjusted using the Benjamini–Hochberg false discovery rate procedure. The preparedness composite represents the sum of 15 self-reported preparedness behaviors and should not be interpreted as a validated psychometric scale. The earthquake-related anxiety composite represents the sum of five exploratory items and is not a diagnostic instrument. For multiple comparisons, a two-sided FDR-adjusted p-value <0.05 was considered statistically significant.

### Preparedness and earthquake-related anxiety across participant characteristics

The preparedness composite differed significantly according to sex, age group, education, income, housing tenure, number of building floors, and building age. These associations remained statistically significant after Benjamini–Hochberg false discovery rate adjustment. Effect sizes were generally small, with the largest values observed for sex (rank-biserial r=0.187) and housing tenure (rank-biserial r=−0.124) (Table 1).

The earthquake-related anxiety composite also differed significantly according to sex, age group, education, income, number of building floors, and building age. In contrast, anxiety values did not differ significantly according to housing tenure (p=0.091; FDR-adjusted p=0.097). Effect sizes for these comparisons were small overall, with the largest values observed for sex (rank-biserial r=−0.137), age group (epsilon-squared=0.115), and education (epsilon-squared=0.086) (Table 1).

### Distribution of reported preparedness behaviors

**Figure 1:**
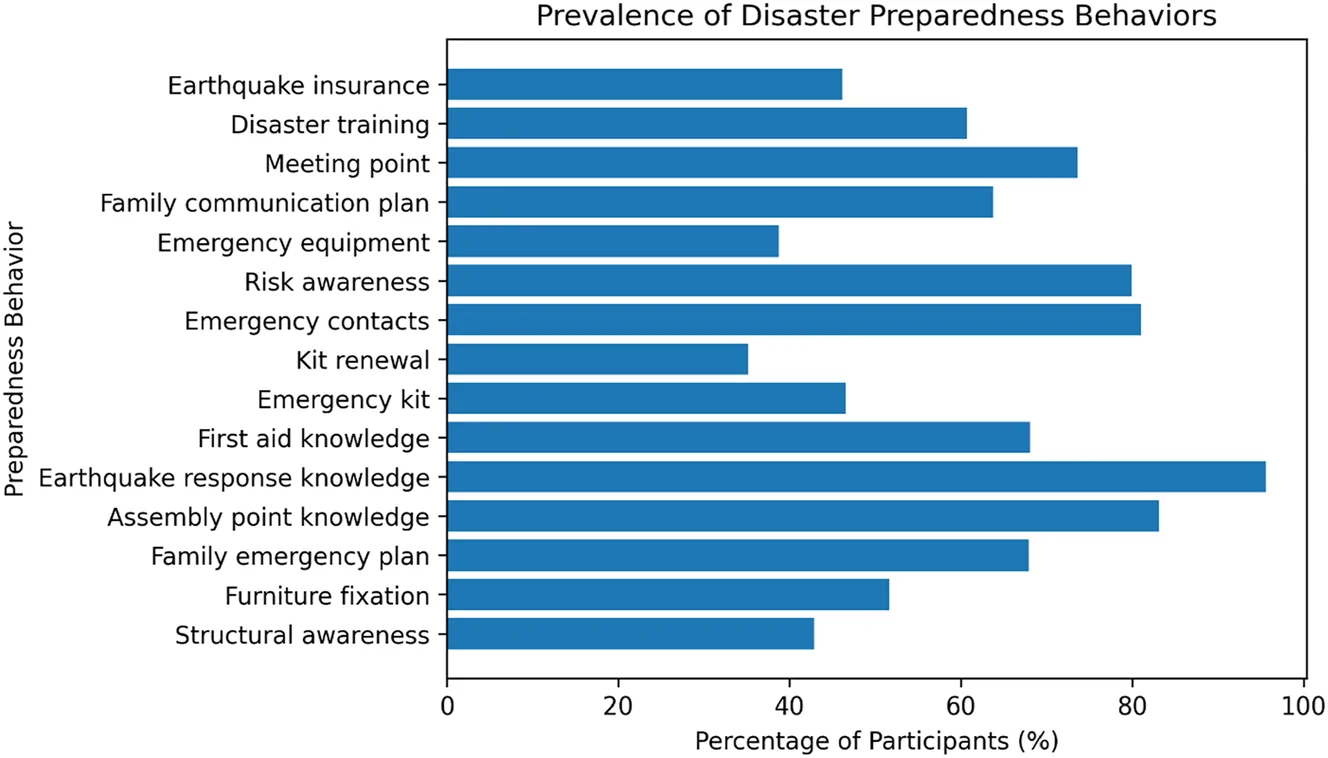
Prevalence of disaster preparedness behaviors among study participants. The figure illustrates the proportion of participants reporting specific disaster preparedness behaviors. Knowledge-based preparedness behaviors, such as correct earthquake response knowledge and awareness of emergency assembly points, were highly prevalent, whereas practical preparedness measures, including regular renewal of disaster kits and availability of emergency equipment, were less common*.*

Knowledge-based preparedness behaviors were reported more frequently than several practical household measures. Knowledge of correct behavior during an earthquake was reported by 95.6 % of participants, knowledge of the local emergency assembly point by 83.1 %, pre-identification of emergency contacts by 81.0 %, and awareness of regional earthquake risk by 79.9 %. In contrast, 46.6 % reported having an emergency disaster kit at home, 38.7 % reported having emergency equipment, and 35.2 % reported regularly renewing the disaster kit. Earthquake insurance coverage was reported by 46.2 % of participants (Table 2; Figure 1).

**Table 2:** Distribution of reported earthquake-preparedness behaviors and their associations with the earthquake-related anxiety composite.

Reported preparedness behavior		Anxiety composite, median (IQR)		p-Value	Effect size	FDR-adjusted p-Value
Behavior	Yes, n (%)	Yes	No		Rank-biserial r	
Knowledge of building earthquake resistance	1,700 (42.9)	12 (6–15)	15 (10–19)	<0.001	−0.289	<0.001
Furniture fixation (cabinets/shelves secured)	2,048 (51.7)	13 (7–17)	14 (10–19)	<0.001	−0.127	<0.001
Family emergency plan	2,694 (68.0)	14 (9–19)	13 (6–18)	<0.001	0.151	<0.001
Knowledge of local emergency assembly point	3,293 (83.1)	13 (7–18)	15 (11–20)	<0.001	−0.253	<0.001
Knowledge of correct behavior during an earthquake	3,789 (95.6)	13 (7–18)	14 (9–15)	0.005	0.126	0.005
First-aid knowledge	2,700 (68.1)	13 (7–17)	15 (10–18)	<0.001	−0.135	<0.001
Emergency disaster kit available at home	1,847 (46.6)	13 (7–18)	14 (10–18)	0.012	−0.046	0.013
Regular renewal of disaster kit	1,396 (35.2)	12 (5–19)	14 (10–18)	<0.001	−0.130	<0.001
Pre-identified emergency contacts	3,210 (81.0)	14 (7–18)	13 (9–18)	0.501	−0.016	0.501
Awareness of regional earthquake risk	3,169 (79.9)	14 (9–18)	10 (5–14)	<0.001	0.320	<0.001
Emergency equipment available at home	1,536 (38.7)	13 (5–16)	14 (9–19)	<0.001	−0.159	<0.001
Family communication plan during a disaster	2,529 (63.8)	13 (7–18)	14 (9–18)	<0.001	−0.084	<0.001
Post-disaster meeting point identified	2,917 (73.6)	13 (7–17)	17 (12–20)	<0.001	−0.280	<0.001
Participation in disaster-preparedness training	2,408 (60.7)	14 (9–18)	12 (7–18)	0.001	0.060	0.002
Earthquake insurance coverage	1,830 (46.2)	13 (10–18)	13 (5–18)	<0.001	0.082	<0.001

Data are presented as n (%) for affirmative responses and median (interquartile range, IQR) for the earthquake-related anxiety composite. Anxiety values were compared between participants reporting and not reporting each preparedness behavior using the Mann–Whitney U test. Effect size is reported as rank-biserial correlation. To account for multiple hypothesis testing across the preparedness items, p values were additionally adjusted using the Benjamini–Hochberg false discovery rate procedure. The preparedness items reflect distinct self-reported behaviors rather than a validated unidimensional scale, and the earthquake-related anxiety composite should be interpreted as an exploratory, non-diagnostic measure. For multiple comparisons, a two-sided FDR-adjusted p-value <0.05 was considered statistically significant.

Earthquake-related anxiety values differed between participants who reported and did not report 14 of the 15 preparedness behaviors after false discovery rate adjustment. Pre-identification of emergency contacts was the only behavior that was not significantly associated with the anxiety composite (p=0.501; FDR-adjusted p=0.501). The largest absolute effect sizes were observed for awareness of regional earthquake risk (rank-biserial r=0.320), knowledge of building earthquake resistance (r=−0.289), identification of a post-disaster meeting point (r=−0.280), and knowledge of the local emergency assembly point (r=−0.253) (Table 2).

Participants who reported awareness of regional earthquake risk had higher anxiety values than those who did not. In contrast, participants reporting knowledge of building resistance, awareness of an emergency assembly point, or identification of a post-disaster meeting point generally had lower anxiety values. Although several other comparisons were statistically significant, their effect sizes were small (Table 2).

### Item-level earthquake-related anxiety responses

**Figure 2:**
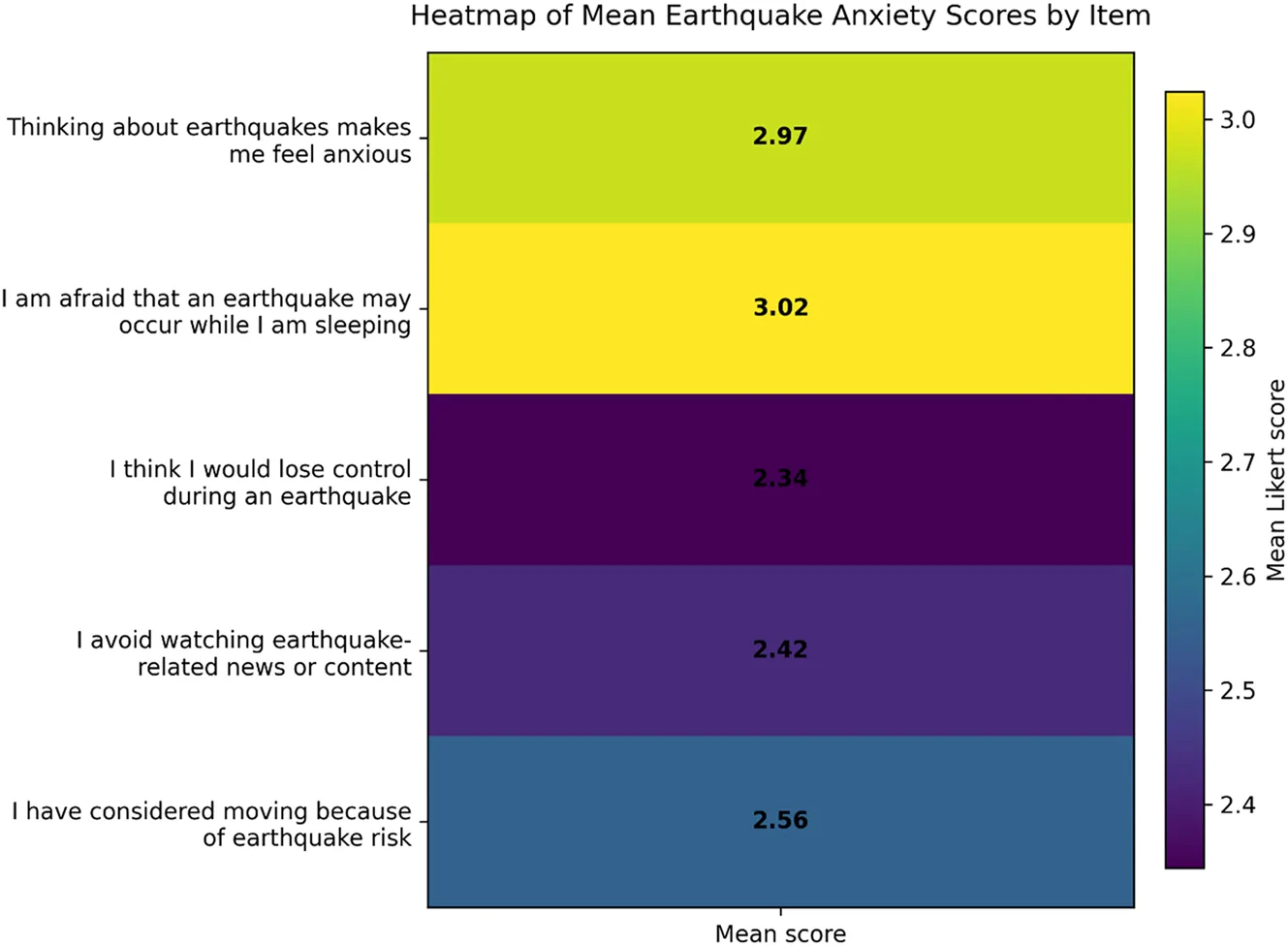
Mean responses to earthquake-related anxiety items. The heatmap presents the mean responses to the five earthquake-related anxiety items. Warmer colors indicate stronger endorsement of the respective item. Fear that an earthquake might occur during sleep and anxiety when thinking about earthquakes had the highest mean responses. Each item was rated on a five-point Likert-type response format ranging from 1 (strongly disagree) to 5 (strongly agree).

Among the five earthquake-related anxiety items, the highest mean response was observed for fear that an earthquake might occur during sleep (3.02 ± 1.58), followed by feeling anxious when thinking about earthquakes (2.97 ± 1.55). The lowest mean response was observed for perceived loss of control during an earthquake (2.34 ± 1.42), followed by avoidance of earthquake-related news or content (2.42 ± 1.50) (Table 3; Figure 2).

**Table 3:** Item-level descriptive distribution of earthquake-related anxiety responses.

Earthquake-related anxiety item	Mean ± SD	Median (IQR)
Thinking about earthquakes makes me feel anxious	2.97 ± 1.55	3 (1–4)
I am afraid that an earthquake may occur while I am sleeping	3.02 ± 1.58	3 (1–5)
I think I would lose control during an earthquake	2.34 ± 1.42	2 (1–3)
I avoid watching earthquake-related news or content	2.42 ± 1.50	2 (1–4)
I have considered moving because of earthquake risk	2.56 ± 1.55	2 (1–4)

Data are presented as mean ± standard deviation (SD) and median (interquartile range, IQR). Each item was rated on a five-point Likert-type response format ranging from 1 (strongly disagree) to 5 (strongly agree), with higher values indicating a stronger earthquake-related anxiety response. These investigator-developed items were used as an exploratory composite measure and should not be interpreted as a validated diagnostic scale or psychiatric instrument.

### Overall composite distributions and correlation

**Figure 3:**
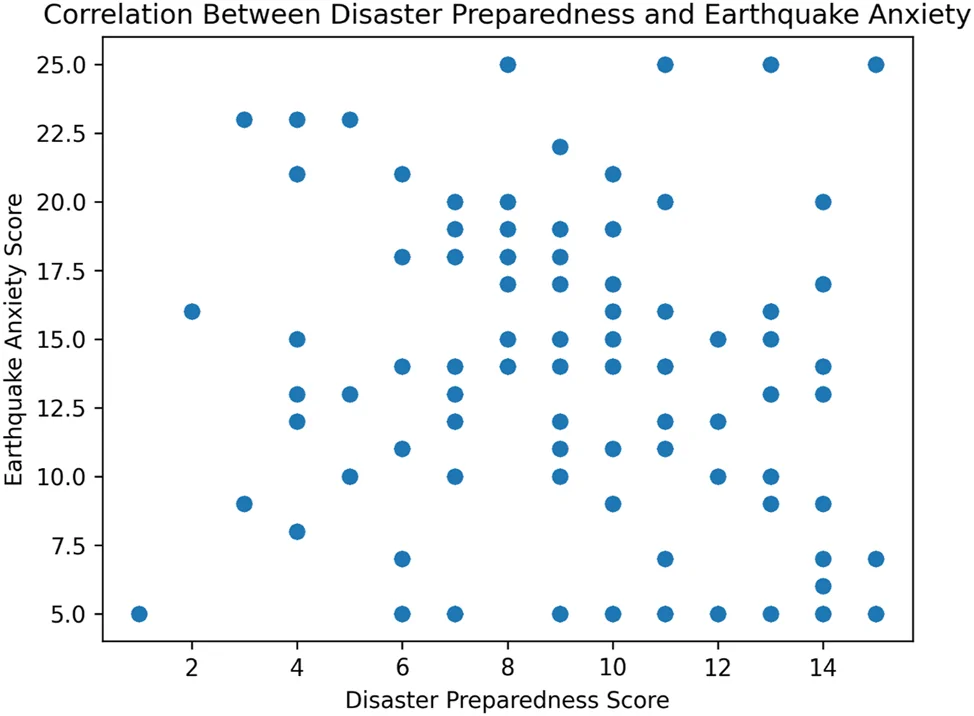
Association between the preparedness behavioral composite and the earthquake-related anxiety composite. The scatter plot illustrates the association between the two continuous composites. A statistically significant but very weak inverse correlation was observed (Spearman’s ρ=−0.107; 95 % CI, −0.140 to −0.070). The plot should not be interpreted as evidence of a causal or clinically important relationship.

The preparedness composite had a mean of 9.35 ± 3.25 and a median of 9 (IQR 7–12). The earthquake-related anxiety composite had a mean of 13.32 ± 6.12 and a median of 13 (IQR 7–18). The two continuous composites showed a statistically significant but very weak inverse correlation (Spearman’s ρ=−0.107, 95 % CI −0.140 to −0.070; p<0.001). Thus, higher preparedness values were associated with slightly lower anxiety values, although the magnitude of the relationship was limited (Table 4; Figure 3).

**Table 4:** Distribution of the preparedness and earthquake-related anxiety composites and their correlation.

Measure	Possible range	Observed range	Mean ± SD	Median (IQR)
Preparedness composite	0–15	1–15	9.35 ± 3.25	9 (7–12)
Earthquake-related anxiety composite	5–25	5–25	13.32 ± 6.12	13 (7–18)

Association between the two continuous composites			
Association	Spearman’s ρ	95 % CI	p-Value
Preparedness composite vs. earthquake-related anxiety composite	−0.107	−0.140 to −0.070	<0.001

The preparedness composite represents the sum of 15 self-reported preparedness behaviors, and the earthquake-related anxiety composite represents the sum of five exploratory Likert-type items. Neither composite should be interpreted as a validated diagnostic or psychometric scale. The 95 % confidence interval for Spearman’s rho was estimated by bootstrap resampling. The correlation was statistically significant but very weak in magnitude. Low, moderate, and high preparedness categories are not presented in this table because they are nonvalidated descriptive cut points; the continuous preparedness composite is used in the primary analyses. A two-sided p-value <0.05 was considered statistically significant.

### Internal consistency and exploratory internal structure

The preparedness behavioral composite demonstrated acceptable internal consistency (Cronbach’s α=0.761). Corrected item–total correlations ranged from 0.087 to 0.550, and α values after deletion of individual items ranged from 0.730 to 0.770. Sampling adequacy was acceptable (KMO=0.726), and Bartlett’s test of sphericity was significant (χ^2^=14,477.42, df=105; p<0.001). Parallel analysis retained five dimensions, which together explained 61.2 % of the variance. Absolute item loadings ranged from 0.446 to 0.827. These findings indicate a multidimensional internal structure consistent with the heterogeneous behavioral content of the preparedness checklist rather than a single psychometric dimension (Table 5).

**Table 5:** Internal consistency and exploratory internal-structure analyses of the preparedness and earthquake-related anxiety composites.

Composite	No. of items	Cronbach’s α	Corrected item–total correlation, range	α if item deleted, range	KMO	Bartlett’s test, χ^2^ (df)	Dimensions retained by parallel analysis	Cumulative variance explained, %	Absolute loading range
Preparedness behavioral composite	15	0.761	0.087–0.550	0.730–0.770	0.726	14,477.42 (105)^a^	5	61.2	0.446–0.827
Earthquake-related anxiety composite	5	0.864	0.588–0.786	0.809–0.859	0.829	9,494.68 (10)^a^	1	65.0	0.723–0.881

KMO, Kaiser–Meyer–Olkin measure of sampling adequacy. ^a^p-Value <0.001 for Bartlett’s test of sphericity. The number of dimensions was determined using parallel analysis. Loading ranges represent the largest absolute loading for each item on the retained dimensions. These analyses evaluate internal consistency and exploratory internal structure within the present sample; they do not establish external validation, diagnostic validity, or a new psychometric scoring system. The preparedness composite comprises distinct self-reported behaviors and demonstrated a multidimensional structure; it should therefore be interpreted as a pragmatic behavioral checklist composite. The earthquake-related anxiety composite showed a single-dimension exploratory structure but should not be interpreted as a diagnostic psychiatric instrument. A two-sided p-value <0.05 was considered statistically significant.

The earthquake-related anxiety composite demonstrated good internal consistency (Cronbach’s α=0.864). Corrected item–total correlations ranged from 0.588 to 0.786, and α-if-item-deleted values ranged from 0.809 to 0.859. Sampling adequacy was good (KMO=0.829), and Bartlett’s test was significant (χ^2^=9,494.68, df=10; p<0.001). Parallel analysis supported a single dimension explaining 65.0 % of the variance, with absolute item loadings ranging from 0.723 to 0.881. These findings support a coherent exploratory internal structure in this sample but do not establish diagnostic or external validity (Table 5).

### Multivariable associations

In the multivariable model with the preparedness composite as the dependent variable, the earthquake-related anxiety composite remained independently associated with a slightly lower preparedness value after adjustment for sex, age group, marital status, household size, housing tenure, occupation, education, monthly income, number of building floors, and building age (B=−0.074, 95 % CI −0.090 to −0.059; p<0.001). The model explained 34.1 % of the variance in the preparedness composite (adjusted R^2^=0.335) (Table 6).

**Table 6:** Multivariable associations with the preparedness and earthquake-related anxiety composites.

Variable	Comparison/unit	Model A: Preparedness composite B (95 % CI)	p-Value	Model B: Anxiety composite B (95 % CI)	p-Value
Sex	Female vs. male	0.132 (−0.103, 0.368)	0.271	3.326 (2.940, 3.712)	<0.001
Age group	30–40 vs. 18–30 years	1.541 (1.251, 1.830)	<0.001	−0.710 (−1.301, −0.119)	0.018
	40–50 vs. 18–30 years	1.145 (0.681, 1.609)	<0.001	3.775 (2.888, 4.662)	<0.001
	50–60 vs. 18–30 years	2.384 (2.000, 2.768)	<0.001	−0.145 (−0.846, 0.556)	0.685
	60–70 vs. 18–30 years	2.663 (2.143, 3.182)	<0.001	0.024 (−0.913, 0.962)	0.959
	≥70 vs. 18–30 years	2.594 (2.055, 3.132)	<0.001	1.066 (−0.370, 2.503)	0.146
Marital status	Single vs. married	0.245 (−0.027, 0.516)	0.078	1.572 (1.186, 1.958)	<0.001
Housing tenure	Owner vs. renter	−0.072 (−0.323, 0.179)	0.572	−0.876 (−1.266, −0.487)	<0.001
Occupation	Construction vs. not working	0.038 (−0.723, 0.798)	0.922	4.092 (3.143, 5.041)	<0.001
	Machine/industry vs. not working	1.949 (1.585, 2.313)	<0.001	−0.409 (−1.046, 0.229)	0.209
	Food/clothing/retail vs. not working	0.248 (−0.124, 0.620)	0.192	−2.032 (−2.713, −1.351)	<0.001
	Education/student vs. not working	3.268 (2.782, 3.753)	<0.001	3.658 (3.048, 4.268)	<0.001
	Courier/transportation vs. not working	1.610 (1.160, 2.060)	<0.001	1.828 (0.773, 2.883)	<0.001
	Cleaning vs. not working	2.823 (2.267, 3.379)	<0.001	5.254 (3.715, 6.793)	<0.001
	Healthcare vs. not working	4.041 (3.645, 4.437)	<0.001	4.658 (3.807, 5.509)	<0.001
	Plumbing/furniture vs. not working	5.513 (5.101, 5.924)	<0.001	7.041 (6.212, 7.871)	<0.001
	Electrical work vs. not working	5.375 (4.872, 5.878)	<0.001	−0.722 (−1.591, 0.147)	0.104
	Civil service/desk vs. not working	2.439 (2.020, 2.858)	<0.001	−0.274 (−1.041, 0.493)	0.484
Education	Primary vs. no formal education	2.731 (2.039, 3.423)	<0.001	−8.874 (−10.509, −7.239)	<0.001
	Secondary vs. no formal education	3.847 (3.202, 4.492)	<0.001	−11.914 (−13.464, −10.365)	<0.001
	High school vs. no formal education	3.288 (2.650, 3.926)	<0.001	−8.578 (−10.165, −6.992)	<0.001
	University vs. no formal education	3.377 (2.728, 4.026)	<0.001	−9.276 (−10.875, −7.678)	<0.001
	Postgraduate vs. no formal education	2.650 (1.767, 3.533)	<0.001	−13.105 (−14.870, −11.340)	<0.001
Monthly income	25,000–50,000 vs. 0–25,000	−0.340 (−0.657, −0.024)	0.035	1.230 (0.706, 1.754)	<0.001
	50,000–75,000 vs. 0–25,000	−1.516 (−2.013, −1.019)	<0.001	3.480 (2.760, 4.201)	<0.001
	75,000–100,000 vs. 0–25,000	0.540 (0.004, 1.076)	0.048	2.455 (1.264, 3.646)	<0.001
	100,000–150,000 vs. 0–25,000	−2.330 (−2.889, −1.771)	<0.001	0.413 (−0.631, 1.456)	0.438
	>150,000 vs. 0–25,000	1.834 (1.210, 2.457)	<0.001	−2.576 (−3.621, −1.532)	<0.001
Household size	Per one-person increase	−0.501 (−0.591, −0.410)	<0.001	0.139 (−0.028, 0.306)	0.102
Building floors	Per one-floor increase	0.105 (0.084, 0.126)	<0.001	0.250 (0.211, 0.290)	<0.001
Building age	Per one-year increase	0.015 (0.005, 0.026)	0.003	0.018 (0.001, 0.034)	0.033
Primary cross-composite association	Per one-point increase	−0.074 (−0.090, −0.059)	<0.001	−0.241 (−0.289, −0.193)	<0.001
Model fit		R^2^=0.341; adjusted R^2^=0.335		R^2^=0.399; adjusted R^2^=0.394	

B values are unstandardized regression coefficients with heteroscedasticity-robust (HC3) 95 % confidence intervals. Model A used the preparedness composite as the dependent variable and included the earthquake-related anxiety composite; Model B used the earthquake-related anxiety composite as the dependent variable and included the preparedness composite. Both models were simultaneously adjusted for sex, age group, marital status, household size, housing tenure, occupation, education, monthly income, number of building floors, and building age. Reference categories are stated in the comparison column. The preparedness measure is a pragmatic composite count of self-reported behaviors, and the anxiety measure is an exploratory non-diagnostic composite. Coefficients represent adjusted associations and should not be interpreted causally. A two-sided p-value <0.05 was considered statistically significant.

Age group, occupation, education, income, household size, number of building floors, and building age were also independently associated with preparedness. Sex, marital status, and housing tenure were not independently associated with preparedness in the fully adjusted model (Table 6).

In the multivariable model with the earthquake-related anxiety composite as the dependent variable, higher preparedness remained independently associated with a slightly lower anxiety value (B=−0.241, 95 % CI −0.289 to −0.193; p<0.001). The model explained 39.9 % of the variance in the anxiety composite (adjusted R^2^=0.394) (Table 6).

Female sex, marital status, housing tenure, several age and occupational categories, education, income, number of building floors, and building age were independently associated with anxiety. Household size was not significantly associated with anxiety (p=0.102). The adjusted cross-composite coefficients were small and represent associations rather than evidence of a causal relationship in either direction (Table 6).

## Discussion

The present study provides a detailed evaluation of self-reported earthquake-preparedness behaviors and earthquake-related anxiety responses among adults presenting to a tertiary emergency department. Several findings merit emphasis. First, knowledge-based preparedness behaviors were commonly reported, whereas implementation of several practical household measures remained limited. Second, preparedness and anxiety values varied across demographic, socioeconomic, occupational, and residential characteristics, although most unadjusted effect sizes were small. Third, the preparedness and anxiety composites were inversely associated in both unadjusted and adjusted analyses, but the magnitude of this relationship was limited. Finally, exploratory internal-structure analyses showed that the preparedness items reflected multiple behavioral dimensions rather than a single psychometric construct, whereas the five anxiety items demonstrated a coherent single-dimension structure within this sample.

The contribution of the present study does not lie in establishing that disaster preparedness is important, which is already well recognized, but in characterizing the awareness–action gap, the heterogeneous internal structure of preparedness behaviors, and the small adjusted association between preparedness and earthquake-related anxiety in a large emergency department sample. These findings add detail to the existing literature by showing that preparedness is not a uniform construct and that statistically significant relationships with psychological responses may nevertheless be modest in magnitude.

### Preparedness patterns and the awareness–action gap

Earthquake preparedness comprises risk awareness, practical knowledge, household planning, access to resources, and the ability to undertake protective actions [2], [3], [4]. In the present study, most participants reported knowing what to do during an earthquake, being aware of the local emergency assembly point, and recognizing regional earthquake risk. In contrast, fewer than half reported having an emergency disaster kit, earthquake insurance, or emergency equipment at home, and only approximately one-third regularly renewed their disaster kit. This discrepancy is consistent with the previously described gap between awareness of risk and implementation of concrete protective behaviors [5], 6].

Behavioral models suggest that awareness alone may be insufficient to generate preparedness. Protective action depends not only on perceived threat but also on whether the recommended action is considered feasible, effective, affordable, and within the individual’s control [2], 3], 7]. Some preparedness behaviors, such as knowing an assembly point, require little financial investment, whereas securing household furniture, maintaining emergency supplies, or obtaining insurance may require economic resources, housing authority, time, and sustained motivation. Therefore, the observed gap should not be interpreted simply as a lack of knowledge. Practical, structural, and socioeconomic barriers may also limit the translation of awareness into action.

A recent large cross-sectional study from Türkiye similarly reported associations between disaster literacy, education, and preparedness behaviors [11]. Household-level research from Iran also demonstrated that preparedness is unequally distributed and related to access to information, resources, and social circumstances [12]. In the present study, multivariable analysis showed that preparedness was associated with several factors simultaneously, including age, occupation, education, income, household size, and residential characteristics. However, these associations were not uniformly graded across all categories. This complexity suggests that preparedness is unlikely to be explained by a single socioeconomic characteristic and may instead reflect the combined influence of knowledge, resources, occupational exposure, perceived vulnerability, housing conditions, and practical opportunity.

The multidimensional internal structure of the preparedness composite supports this interpretation. Behaviors related to knowledge, planning, equipment, training, communication, and financial protection may arise from different mechanisms. Accordingly, a participant may report strong preparedness in one domain but limited action in another. This heterogeneity is important when interpreting total preparedness values and argues against treating the composite as a single latent psychological trait.

### Preparedness behaviors and earthquake-related anxiety

The item-level associations between preparedness behaviors and anxiety were not uniformly directional. Participants who reported knowing whether their residence was earthquake resistant, knowing an emergency assembly point, or identifying a post-disaster meeting point generally had lower anxiety values. These behaviors may be associated with clearer response plans, greater practical knowledge, or a stronger sense of control. In contrast, awareness of regional earthquake risk showed the largest positive association with anxiety, and some planning- or training-related behaviors were also associated with higher anxiety values.

Protection motivation theory proposes that protective behavior may arise from the combined appraisal of threat and coping capacity [13]. Greater concern may encourage some individuals to seek information, attend training, or undertake selected preparedness actions, whereas practical knowledge and confidence may coexist with lower distress. Evidence from Japan likewise indicates that perceived risk and previous disaster experience are associated with household preparedness, although the relationship between concern and action is not necessarily linear [14]. The mixed direction of the item-level findings in the present study is therefore plausible and cautions against interpreting all preparedness behaviors as consequences of either low or high anxiety.

It is also important that several statistically significant item-level differences had small effect sizes. With a sample of nearly 4,000 participants, modest differences may generate small p values. Although false discovery rate adjustment did not materially change the overall pattern, the effect-size estimates indicate that statistical significance should not be equated with substantial psychological or practical importance.

### Earthquake-related psychological responses

Among the five anxiety-related items, fear that an earthquake might occur during sleep and anxiety when thinking about earthquakes received the highest mean responses. These findings are compatible with the anticipatory and uncertainty-related responses described in disaster mental-health research [8]. Studies conducted after the 2023 Kahramanmaraş earthquakes have also documented anxiety, sleep disturbance, and psychological distress among exposed populations and healthcare workers [15].

However, the present earthquake-related anxiety composite was investigator-developed and was not a diagnostic psychiatric instrument. The findings should therefore not be interpreted as the prevalence of an anxiety disorder or as evidence of clinically significant anxiety. The item responses describe earthquake-related concerns within this sample rather than a formal psychiatric diagnosis.

The five anxiety items demonstrated good internal consistency and a coherent single-dimension exploratory structure. Nevertheless, internal coherence in one dataset does not establish external, convergent, discriminant, criterion, or diagnostic validity. Because no external criterion measure or previously validated earthquake-specific anxiety instrument was administered, it was not possible to determine how closely this composite corresponded to established measures of anxiety, fear, risk perception, or psychological preparedness.

### Relationship between preparedness and anxiety

The preparedness and anxiety composites showed a statistically significant inverse correlation, but the magnitude was very weak. The adjusted models similarly indicated that higher preparedness was associated with slightly lower anxiety and that higher anxiety was associated with slightly lower preparedness after controlling for demographic, socioeconomic, occupational, and residential characteristics. These findings indicate that the association persisted after covariate adjustment but do not support a large or clinically important relationship.

Several explanations are possible. Preparedness may be associated with greater perceived control, clearer expectations, and reduced uncertainty. Previous work has suggested that perceived control and response confidence may contribute to psychological adaptation in disaster-prone settings [16]. Conversely, individuals with greater earthquake-related concern may be more likely to seek information, attend training, or undertake selected preparedness behaviors. At higher levels, concern may also coexist with avoidance, helplessness, or fatalistic responses. Residual confounding and the shared use of self-reported data may also have contributed to the observed relationship.

The cross-sectional design precludes determination of temporal direction. It is therefore not possible to conclude that preparedness reduces anxiety, that anxiety inhibits preparedness, or that either construct causes a change in the other. The reciprocal regression models should be interpreted as complementary adjusted descriptions rather than as competing causal models. The small coefficients and very weak correlation further support a cautious interpretation.

### Internal structure of the investigator-developed composites

The exploratory internal-structure findings clarified that the preparedness and anxiety composites should not be interpreted in the same way. The 15 preparedness items covered building awareness, household safety, emergency planning, communication, equipment, training, and financial protection. Parallel analysis retained five dimensions, consistent with the heterogeneous content of these behaviors. The total preparedness value is therefore best understood as a pragmatic count of reported actions and knowledge-related practices rather than as a unidimensional psychometric scale.

The low corrected item–total correlation observed for some preparedness items is also compatible with this multidimensionality. For example, earthquake insurance may be strongly influenced by home ownership and financial circumstances, whereas knowledge of an assembly point may depend primarily on access to information. These behaviors need not correlate strongly even though both are relevant to preparedness. Accordingly, Cronbach’s alpha alone should not be used to claim validation of a new preparedness scale.

In contrast, the five anxiety items showed stronger corrected item–total correlations, good internal consistency, and a single-dimension exploratory structure. These findings support the use of their sum as an exploratory descriptive composite in the present dataset. However, they do not establish test–retest reliability, criterion validity, diagnostic validity, or external generalizability.

### Emergency department perspective

Emergency departments provide access to individuals with varied demographic and socioeconomic backgrounds and may therefore be useful settings for identifying preparedness gaps among healthcare-seeking populations [9], 10]. However, emergency department attendees should not be considered representative of the general community. Individuals presenting for emergency care may differ from the broader population in health status, stress level, time availability, literacy, and willingness to participate in research.

The large sample nevertheless provides a detailed description of preparedness behaviors in an emergency department population. Brief educational interventions have been associated with improvements in disaster knowledge and selected preparedness behaviors in other populations [17]. Psychological preparedness and response self-efficacy have also been linked to disaster-risk perception among healthcare-related groups [18]. In addition, studies among emergency healthcare workers have demonstrated substantial variability in preparedness and confidence, indicating that preparedness cannot be assumed even within healthcare environments [19]. Evidence from training studies and systematic reviews suggests that structured educational approaches may improve disaster-related knowledge and competence, although the durability and generalizability of these effects vary [20]. Recent evidence examining referral patterns across primary, secondary, and tertiary healthcare levels further indicates that resource availability and referral-system organization can substantially shape patient flow toward higher-level facilities [21].

Emergency departments may therefore offer selected opportunities for brief, practical, and non-disruptive preparedness messaging. Such interventions should not interfere with acute care and should be evaluated prospectively for feasibility, acceptability, and effectiveness. The present cross-sectional study does not establish that emergency-department education would improve preparedness or reduce anxiety.

### Public health implications

The findings suggest that preparedness initiatives should move beyond general risk awareness and emphasize feasible household actions. Communication strategies may be more useful when they combine information about risk with practical guidance on emergency kits, family communication, meeting points, structural safety, and access to local resources. Because awareness of regional earthquake risk was associated with higher anxiety, risk communication should avoid relying solely on threat amplification and should incorporate self-efficacy, coping guidance, and realistic actions that individuals can undertake [7], 13].

Preparedness strategies may also require adaptation to educational, occupational, economic, and housing circumstances. Renters may have limited authority to make structural changes, lower-income households may face difficulty obtaining supplies or insurance, and some occupational groups may have greater access to safety training than others. A uniform educational message may therefore be less effective than interventions tailored to the specific barriers faced by different groups.

### Strengths and limitations

This study has several strengths. It included a large sample, assessed a broad range of individual preparedness behaviors, reported effect sizes and confidence intervals, controlled for multiple testing, and used multivariable models to evaluate adjusted associations. The revision also incorporated corrected item–total correlations, alpha-if-item-deleted analyses, sampling-adequacy measures, and exploratory internal-structure assessment rather than relying on Cronbach’s alpha alone.

Several limitations must nevertheless be considered. First, the study was conducted in a single tertiary emergency department using convenience sampling. The findings cannot be generalized directly to the general population, other geographic regions, or other healthcare settings. Emergency department attendees may differ from the broader population in health status, perceived urgency, stress, literacy, time availability, and willingness to participate.

Second, the exact number and sociodemographic characteristics of eligible individuals who declined participation or discontinued the questionnaire were not systematically recorded. Some eligible individuals declined because they did not wish to remain in the emergency department longer, had time constraints or urgent personal obligations, were unwilling to complete the questionnaire, or stopped before completion. Consequently, participants and nonparticipants could not be compared, and nonresponse bias could not be quantified. Individuals with lower educational attainment, lower disaster knowledge, less interest in preparedness, or greater difficulty completing written forms may have been underrepresented, although the direction and magnitude of this potential bias are unknown.

Third, all preparedness behaviors and earthquake-related anxiety responses were self-reported. The data may therefore have been affected by recall error, misunderstanding of questions, or social-desirability bias. Some participants may have overreported desirable preparedness behaviors, while others may have interpreted individual items differently.

Fourth, both composites were based on investigator-developed items. The preparedness composite demonstrated a multidimensional structure and should be interpreted as a behavioral checklist count rather than as a validated psychometric scale. The anxiety composite showed a coherent exploratory internal structure, but no external criterion measure was available. Therefore, convergent, discriminant, criterion, and diagnostic validity could not be assessed. Test–retest reliability was also not evaluated.

Fifth, the original low, moderate, and high preparedness categories were based on nonvalidated divisions of the possible range. These categories were not used in the primary analyses, which relied on the continuous preparedness composite. Any descriptive use of these categories should therefore be interpreted cautiously.

Sixth, no formal *a priori* sample-size calculation was performed. The final sample reflected eligible participants who completed the survey during the predefined recruitment period. The large sample provided precise estimates but also increased the likelihood that small differences would reach statistical significance. For this reason, effect sizes, confidence intervals, and false discovery rate-adjusted p values were emphasized.

Seventh, although the multivariable models included a broad set of demographic, socioeconomic, occupational, and residential covariates, residual and unmeasured confounding remain possible. Potentially relevant factors such as previous earthquake exposure, direct disaster loss, prior psychiatric history, current clinical diagnosis, media exposure, perceived building safety, and prior disaster education outside formal training were not fully characterized.

Eighth, the reciprocal regression models do not establish causal direction. Modeling preparedness as an outcome in one analysis and anxiety as an outcome in another provides complementary descriptions of adjusted associations but cannot resolve whether preparedness influences anxiety, anxiety influences preparedness, or both are shaped by other factors.

Finally, the cross-sectional design prevents assessment of changes over time. Longitudinal studies are needed to determine whether preparedness behaviors are stable, whether earthquake-related anxiety changes after education or disaster exposure, and whether changes in one domain precede changes in the other.

## Conclusions

In this emergency department sample, earthquake-related awareness and knowledge were common, whereas several practical household preparedness behaviors were reported less frequently. Preparedness and earthquake-related anxiety were associated with demographic, socioeconomic, occupational, and residential characteristics. The inverse association between the two composites was statistically significant but small, and the cross-sectional design precludes causal interpretation.

The preparedness measure should be regarded as a multidimensional behavioral checklist composite rather than as a validated psychometric scale. The earthquake-related anxiety composite should likewise be considered exploratory and non-diagnostic. Future multicenter studies using probability-based sampling, externally validated psychological instruments, and prospective designs are needed to confirm these associations, assess temporal relationships, and evaluate whether practical preparedness interventions influence behavior or psychological responses.
